# Supplementary material for: The Construction and Exploration of a Comprehensive MicroRNA Centered Regulatory Network in Foxtail Millet (Setaria italica L.)
Source: Front Plant Sci. 2022 May 6;13:848474. doi: 10.3389/fpls.2022.848474 (PMC9121102; doi:10.3389/fpls.2022.848474)
Supplement: Supplementary file 5 [file Data_Sheet_5.zip › Sit-miRN3020_Seita.9G517900.1_770_TPlot.pdf]

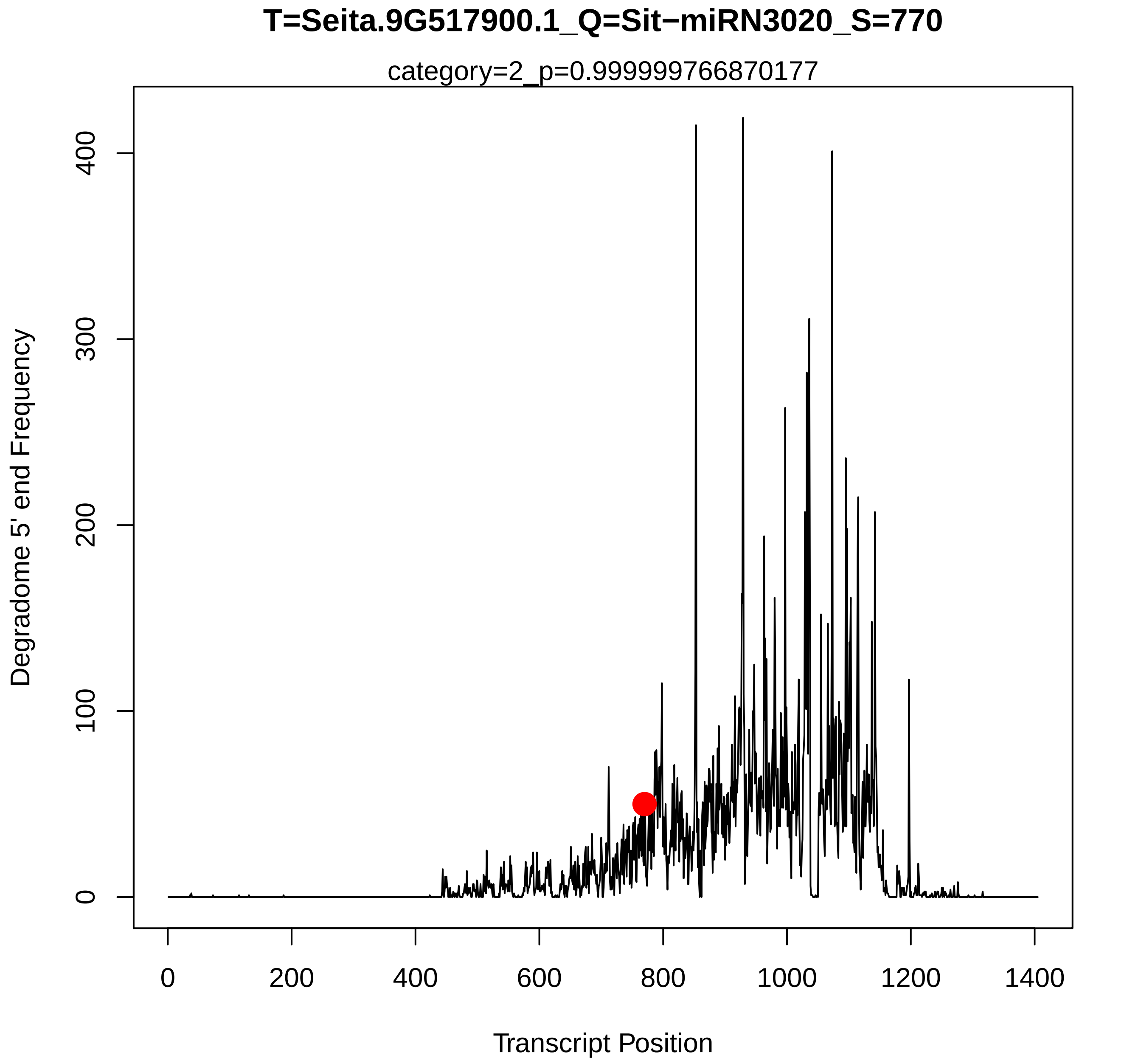

T=Seita.9G517900.1_Q=Sit−miRN3020_S=770
category=2_p=0.999999766870177
Degradome 5' end Frequency
0
100
200
300
400
0
200
400
600
800
1000
1200
1400
Transcript Position
